# The first complete mitochondrial genome of *Murex* from *Murex trapa* (Neogastropoda: Muricidae)

**DOI:** 10.1080/23802359.2019.1674726

**Published:** 2019-10-07

**Authors:** Shengping Zhong, Lianghua Huang, Guoqiang Huang, Yonghong Liu, Weixing Wang

**Affiliations:** a Institute of marine drugs, Guangxi University of Chinese Medicine, Nanning, Guangxi, China;; b Key Laboratory of Marine Biotechnology, Guangxi Institute of Oceanology, Beihai, Guangxi, China;; c School of Artificial Intelligence, The Open University of Guangdong, Guangzhou, Guangdong, China

**Keywords:** Mitochondrial genome, *Murex trapa*, Neogastropoda

## Abstract

The rare-spined murex (*Murex trapa*) is an ecologically and economically important species of Muricidae, which comprises a highly diverse group of predatory marine snails. However, the taxonomic classification and phylogenetic studies have so far been limited. In this study, we report the first complete mitochondrial genome of *Murex* from *M. trapa*. The mitogenome has 15,408 bp (66.3% A + T content) and made up of total of 37 genes (13 protein-coding, 22 transfer RNAs, and 2 ribosomal RNAs), and a control region. This study provided the first complete mitogenome of *Murex* and will provide useful genetic information for future phylogenetic and taxonomic classification of Muricidae.

The Muricidae are one of the most species-rich and morphologically diverse families in the Neogastropoda, which comprise over 1600 described species and inhabited worldwide from intertidal zone to deep sea (Barco et al. 2010). As predators of barnacles, bivalves and other invertebrates, muricids play an ecologically important role in maintaining marine benthic ecosystem (Barco et al. 2010; Zou et al. 2011). Moreover, some muricids including *Murex trapa* are economically important as valuable ingredients of traditional medicines and valuable nutrition of delicious seafood (Benkendorff et al. 2015; Sung et al. 2016). However, the taxonomy and phylogeny of the Muricidae have been in a state of confusion due to the morphological convergence and plasticity (Zou et al. 2011). The complete mitochondrial genome is an excellent molecular marker for studying phylogenetic relationships and taxonomy identification, but adequate mitogenome information about the Muricidae is still limited (Zou et al. 2011). Here, we report the first complete mitochondrial genome sequence of *Murex*, which will provide a better insight into phylogenetic assessment and taxonomic classification.

A tissue samples of *M. trapa* from 5 individuals were collected from GuangXi province, China (Beihai, 21.107303 N, 109.143778 E), and the whole body specimen (#GR0259) were deposited at Marine Biological Herbarium, Guangxi Institute of Oceanology, Beihai, China. The total genomic DNA was extracted from the muscle of the specimens using an SQ Tissue DNA Kit (OMEGA, Guangzhou, China) following the manufacturer’s protocol. DNA libraries (350 bp insert) were constructed with the TruSeq NanoTM kit (Illumina, San Diego, CA) and were sequenced (2 × 150 bp paired-end) using HiSeq platform at Novogene Company, China. Mitogenome assembly was performed by MITObim (Hahn et al. 2013). The complete mitogenome of *Bolinus brandaris* (GenBank accession number: NC_013250) was chosen as the initial reference sequence for MITObim assembly. Gene annotation was performed by MITOS (Bernt et al. 2013).

The complete mitogenome of *M. trapa* was 15,408 bp in length (GenBank accession number: MN462589), and containing the typical set of 13 protein-coding, 22 tRNA and 2 rRNA genes, and a putative control region. The overall base composition of the mitogenome was estimated to be A 28.5%, T 37.8%, C 15.6%, and G 18.0%, with a high A + T content of 66.3%, which is similar, but slightly lower than *Indothais lacera* (68.1%) (Zhong et al. 2017). The mitogenomic phylogenetic analyses showed that *Murex* was first clustered with *Bolinus* then clustered with *Chicoreus* in a single clade with high bootstrap value (Figure 1), which is consistent with the phylogenetic analyses of Muricidae using nuclear gene (*18S rRNA* and segment histone H3) and mitochondrial gene (*COI*, *12S rRNA*, and *16S rRNA*) (Zou et al. 2011). Further phylogenetic studies for Muricidae are needed in future work. The complete mitochondrial genome sequence of *M. trapa* was the first sequenced mitogenome in *Murex*, which will contribute to further phylogenetic and comparative mitogenome studies of Muricidae, and related families.

**Figure 1.**
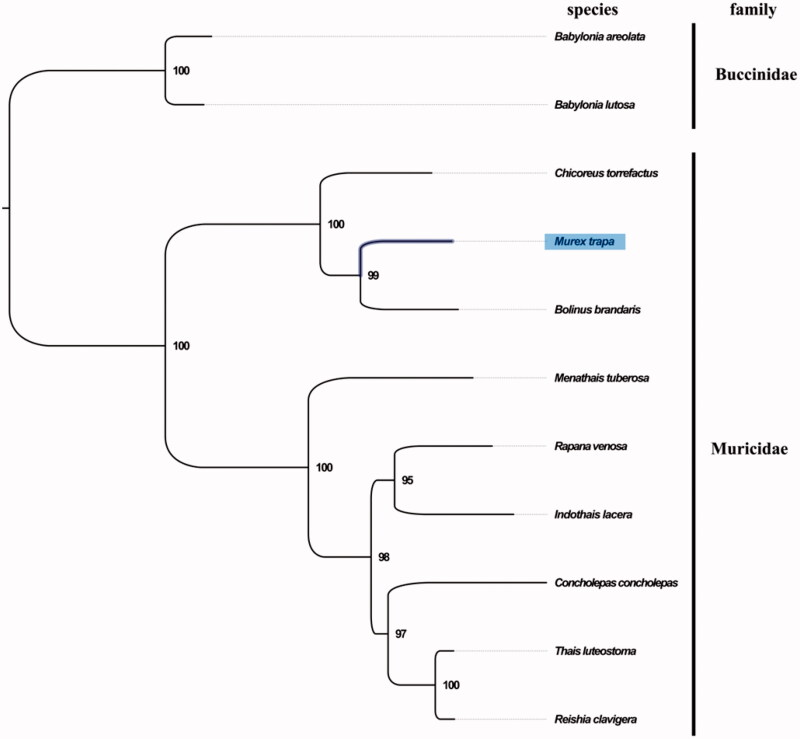
Phylogenetic tree of 11 species in order Neogastropoda. The complete mitogenomes is downloaded from GenBank and the phylogenic tree is constructed by maximum-likelihood method with 100 bootstrap replicates. The bootstrap values were labelled at each branch nodes. The gene’s accession number for tree construction is listed as follows: *Babylonia areolata* (NC_023080), *Babylonia lutosa* (NC_028628), *Chicoreus torrefactus* (NC_039164), *Bolinus brandaris* (NC_013250), *Menathais tuberosa* (NC_031405), *Concholepas concholepas* (NC_017886), *Thais luteostoma* (NC_039165), *Reishia clavigera* (NC_010090), *Rapana venosa* (NC_011193), and *Indothais lacera* (NC_037221).
